# Heroic Saves in Endodontics: Management of Advanced External Cervical Resorption With Intentional Replantation: A Review and Report of Two Cases

**DOI:** 10.1155/crid/5098467

**Published:** 2026-02-26

**Authors:** Mohsen Aminsobhani, Pegah Sarraf, Mehrfam Khoshkhounejad, Maryam Babaahmadi

**Affiliations:** ^1^ Department of Endodontics, Dental Research Center, AJA and Tehran University of Medical Sciences, Tehran, Iran; ^2^ Department of Endodontics, School of Dentistry, Tehran University of Medical Sciences, Tehran, Iran, tums.ac.ir; ^3^ Dental Research Center, Dentistry Research Institute, Tehran University of Medical Sciences, Tehran, Iran, tums.ac.ir

**Keywords:** dental traumatic injuries, intentional replantation, regenerative endodontics, root resorption, tooth avulsion

## Abstract

External cervical resorption (ECR) is a complex and aggressive form of root resorption that poses significant challenges in clinical management. This case report describes two cases of advanced ECR treated with intentional replantation (IR), highlighting its efficacy as a viable treatment option for preservation of compromised teeth. The first case involved a 10‐year‐old girl with a Heithersay Class IV/Patel 3Cd lesion following dental trauma, and the second case involved a 30‐year‐old male with a Heithersay Class IV/Patel 3Bd lesion. Both cases were managed with IR, which included atraumatic extraction, extraoral restoration of defect, and reimplantation within 15 min. Postoperative follow‐ups demonstrated short‐term functional preservation and temporary arrest of resorptive activity; however, long‐term outcomes could not be assessed due to limited follow‐up. The discussion emphasizes the importance of early diagnosis, multidisciplinary collaboration, and adherence to microsurgical principles to optimize the success of IR. Additionally, the utility of Heithersay and Patel’s classification systems in guiding treatment decisions is underscored. These cases illustrate that even in severe ECR, strategic surgical intervention can achieve favorable results, offering clinicians a valuable alternative to extraction.

## 1. Introduction

Root resorption is characterized by the loss of hard tooth structure due to physiological or pathological processes. Physiological root resorption is a natural phenomenon in primary teeth; however, resorption can be detrimental in permanent teeth, potentially leading to tooth loss if left untreated [1, 2]. Root resorption can be classified into internal and external resorption defects, based on its location relative to the root surface. External root resorption (ERR) may be further categorized into several subtypes, including surface resorption, external inflammatory resorption, replacement resorption, external cervical resorption (ECR), and transient apical breakdown. ECR remains one of the least understood forms of ERR. This condition has been extensively described by Heithersay [3–5], who favored the term invasive cervical resorption—a designation that underscores its aggressive and infiltrative behavior.

Cervical root resorption accounts for approximately 4% of all ERR defects [6]. ECR is a distinct form of external resorption primarily affecting the periodontal ligament (PDL), cementum, and dentin, typically occurring at or below the cervical epithelium [3, 7]. The clinical appearance of ECR varies from gingival resorptive defects to pink coronal discoloration, often remaining asymptomatic until pulpal or periodontal involvement occurs [1]. Early detection of ECR remains challenging due to its subgingival location, often leading to incidental radiographic identification and misdiagnosis as root caries [2, 7, 8]. In vital teeth, pulpal involvement is rare unless the lesion is extensive, distinguishing ECR from external inflammatory resorption, where pulp necrosis or infection is definitely present. Resorptive lesions may initially present as a small area of activity on the root surface which can expand coronally, apically, or circumferentially within dentin, often sparing the pulp due to protective predentin until advanced stages, where significant loss of tooth structure occurs [8]. According to histopathological analysis, ECR originates from PDL disruption, triggering inflammatory infiltration, granulation tissue formation, and multinucleated osteoclast‐like cell‐mediated dentin resorption [9]. The lesion progresses circumferentially and apicocoronally, creating resorption channels, while partial repair occurs via osseous‐like tissue deposition. The resorptive tissue comprises vascularized fibrous tissue, fibroblasts, endothelial cells, and leukocytes [10, 11]. The etiology of ECR remains unknown. Several factors have been proposed that could damage the cervical region of the tooth and consequently initiate ECR, including predisposing factors like trauma, orthodontic treatment, parafunctional habits, occlusal dysfunction, coronal bleaching, poor oral hygiene, periodontal therapy, developmental disorders, and viral infections [1, 11]; systemic diseases such as bone metabolism disorders [11, 12], celiac disease (vitamin D3 deficiency), and hypothyroidism [13, 14]; some medications like bisphosphonates, chemotherapeutic agents, and tetracyclines [15, 16]; and idiopathic causes [17]. Definite diagnosis of ECR relies on radiographic findings due to its nonspecific clinical presentation, with features ranging from well‐defined to irregular radiolucencies, reflecting its progressive nature [1]. While conventional radiographs often miss ECR due to overlapping structures, cone‐beam computed tomography (CBCT) provides superior three‐dimensional detection but necessitates careful use to balance diagnostic efficacy with radiation safety [7].

While the existing classification systems serve as valuable tools, the severity and location of the resorption—along with relevant patient‐specific factors—should remain the cornerstone of treatment selection [3, 7]. The Heithersay classification is the conventional method for classification of ECR [18]. It utilizes plain film radiographs to categorize two‐dimensional infiltration of resorption along the root into four distinct classes (Table 1) [7, 18]. However, with the increasing adoption of CBCT in dentistry, the Heithersay’s two‐dimensional classification system is being challenged by a novel three‐dimensional classification proposed by Patel. This new system evaluates the height of the lesion, circumferential extension, and proximity to the root canal using both periapical radiography and CBCT, as shown in Table 2 [19].

**Table 1:** Heithersay’s classification of ECR (invasive cervical resorption).

Class I	A small invasive resorptive lesion near the cervical area with shallow penetration into dentin
Class II	A well‐defined invasive resorptive lesion that has penetrated close to the coronal pulp chamber but shows little or no extension into the radicular dentin
Class III	A deeper invasion of dentin by resorbing tissue, not only involving the coronal dentin but also extending into the coronal third of the root
Class IV	A large, invasive resorptive process that has extended beyond the coronal third of the root

**Table 2:** Patel’s 3D classification of ECR.

Height	Circumferential spread	Proximity to the root canal
1: At the cementoenamel junction level or coronal to the bone crest (supracrestal)	A: ≤90^°^	d: Lesion confined to the dentin
2: Extends into the coronal third of the root and apical to the bone crest (subcrestal)	B: >90^°^ to ≤180^°^	p: Probable pulpal involvement
3: Extends into the midthird of the root	C: >180^°^ to ≤ 270^°^	—
4: Extends into the apical third of the root	D: >270^°^	—

In recent years, multiple cases of ECR have been reported, exhibiting lesions of varying sizes and stages, all successfully managed with the recommended treatments [20, 21]. Intentional replantation (IR) is indicated when orthograde treatment or apical surgery is not an option, as in complex/blocked canals, overfilled roots, contraindicated surgery, external resorption, root perforation/fracture, or developmental anomalies. Recently, IR was proven effective for advanced ECR, particularly in anterior teeth with significant palatal damage. To ensure success, IR must minimize PDL trauma and may combine surgical extrusion with adjunctive procedures for optimal restorative outcomes [22, 23]. It has been reported that small resorption lesions and those located in accessible regions have the most favorable prognosis [24].

This case report describes two cases of advanced ECR treated with IR, highlighting its role as a palliative or temporizing treatment option in carefully selected cases where definitive therapy is not feasible.

## 2. Case Presentation

### 2.1. Case 1

A 10‐year‐old girl was referred to the Endodontics Department of the Faculty of Dentistry, following a diagnosis of root resorption in tooth number 9 (left maxillary central incisor). The referral was prompted by a history of dental trauma 20 months prior. Her medical history was noncontributory, and she reported no pain or discomfort. Her chief complaint included routine dental examination and post‐traumatic follow‐up. According to the referring dentist, tooth #9 had been avulsed due to trauma and had undergone long‐term calcium hydroxide therapy. Following confirmation of apical barrier formation, tooth #9 had been obturated with gutta‐percha using lateral compaction technique. Tooth #10 (left maxillary lateral incisor) had been intruded during the same incident. Due to the absence of spontaneous eruption after 4 months, it had been surgically repositioned using dental forceps. All the abovementioned procedures had been performed prior to her referral to our department.

On clinical examination, no pathological mobility was observed in the affected teeth or adjacent teeth. The palpation and percussion tests revealed no tenderness. Pulp sensibility tests for both teeth showed a negative response to both thermal (cold test) and electrical stimulation tests compared to adjacent teeth. Periodontal probing depth of tooth #9 was <3 mm on the buccal side and 4 mm on the distal‐palatal aspect, without bleeding. Root caries was ruled out as no infected dentin was detected. Radiographic findings at the time of trauma and 20 months post‐trauma are presented in Figure 1. Intraoral radiographs revealed ECR on the distal surface of tooth #9 and incomplete root formation in tooth #10 (Figure 1B); however, no obvious radiolucency was evident at the root apex. Also, there was no evidence of a sinus tract or swelling in the region. CBCT was requested to determine the nature and severity of the resorptive defect. Sagittal and axial CBCT views of tooth #9 demonstrated that the resorptive lesion extended from one‐third to one‐half of the root length, and was classified as Heithersay Class IV lesion [4, 18], affecting both the distal and palatal surfaces, and Class 3Cd lesion according to the Patel’s classification (Figure 2A,B). The sagittal view of tooth #10 revealed less apical development compared to the lateral incisor on the contralateral (right) side (Figure 2C). A multidisciplinary team, including an endodontist, an orthodontist, and a pediatric dentist formulated the treatment plan. Considering the patient’s age, it was decided to retain tooth #9, due to residual vertical growth and to help maintain space; thus, IR was selected to preserve esthetics and promote bone formation. For tooth #10, considering its stage of root development, thin dentinal walls, and wide‐open apex, a regenerative endodontic procedure (REP) was planned. The treatment options were discussed with the patient and her parents, and informed consent was obtained from them for the agreed‐upon procedures.

Figure 1Radiographic examination of teeth #9 and #10. (A) Periapical radiograph taken immediately after dental trauma and repositioning of tooth #9. (B) Periapical radiograph of the same teeth at the 20‐month follow‐up.

**Figure:**
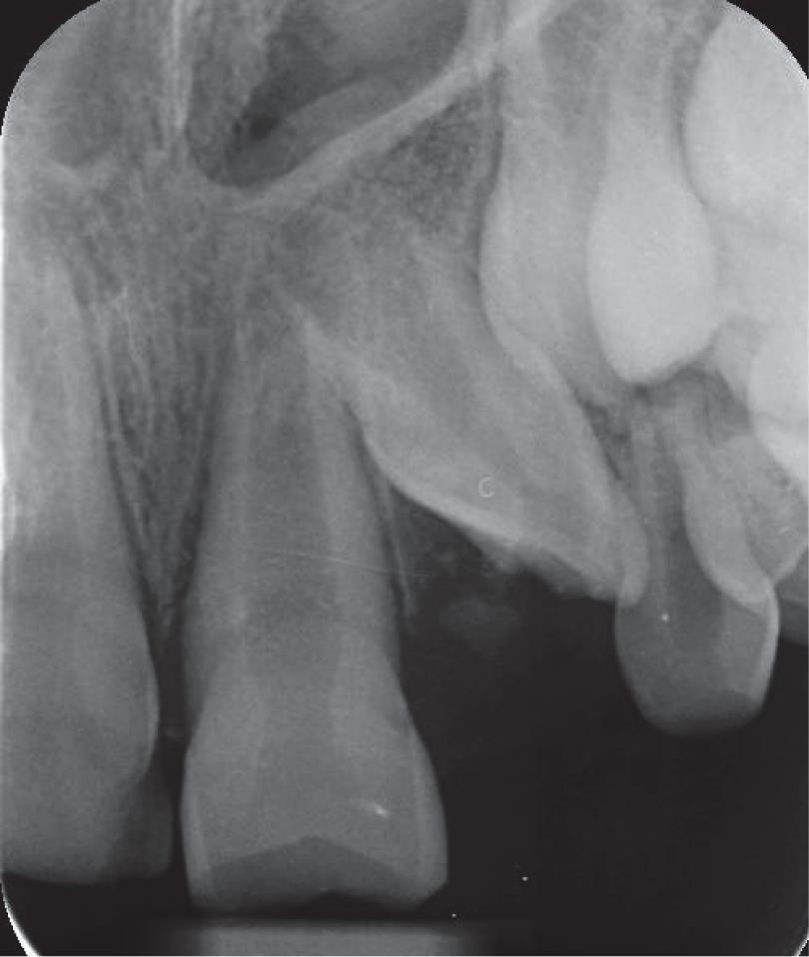
(A)

**Figure:**
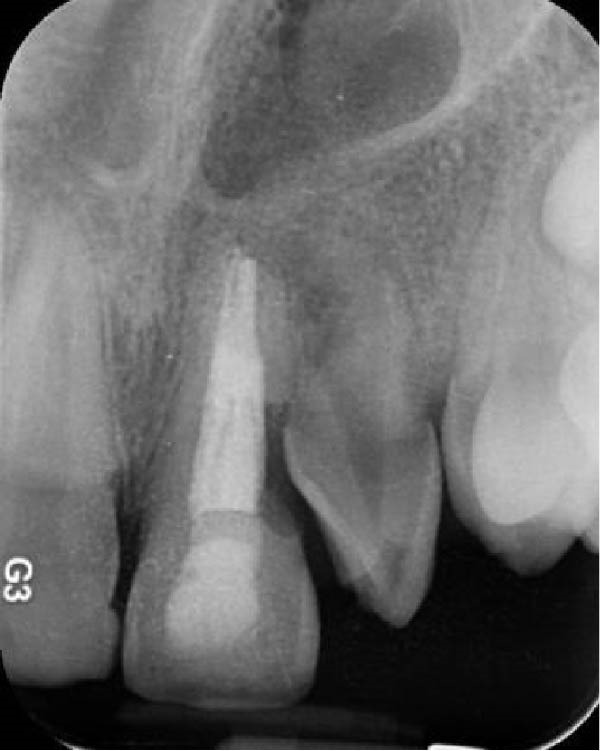
(B)

Figure 2Preoperative CBCT images. (A, B) Sagittal and axial CBCT views of tooth #9, demonstrating ECR. (C) Sagittal view of tooth #10 with incomplete root development and an open apex. (D) Axial view of tooth #10.

**Figure:**
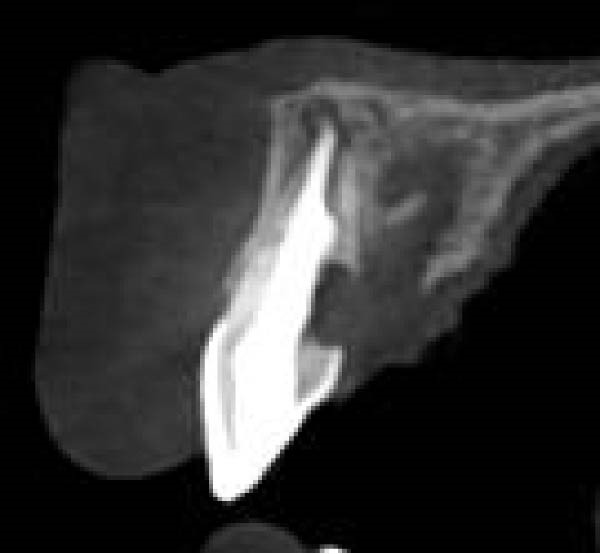
(A)

**Figure:**
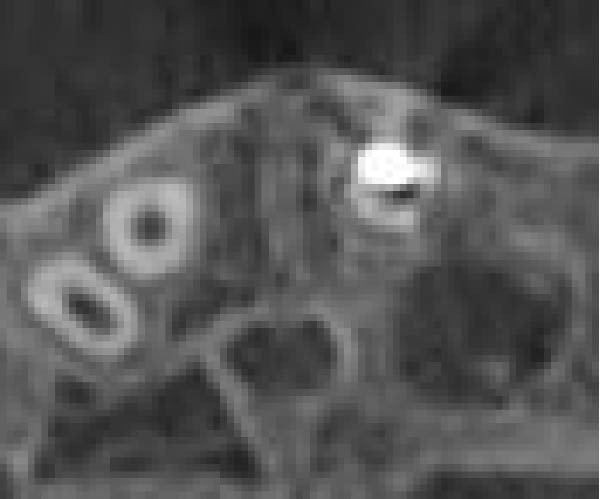
(B)

**Figure:**
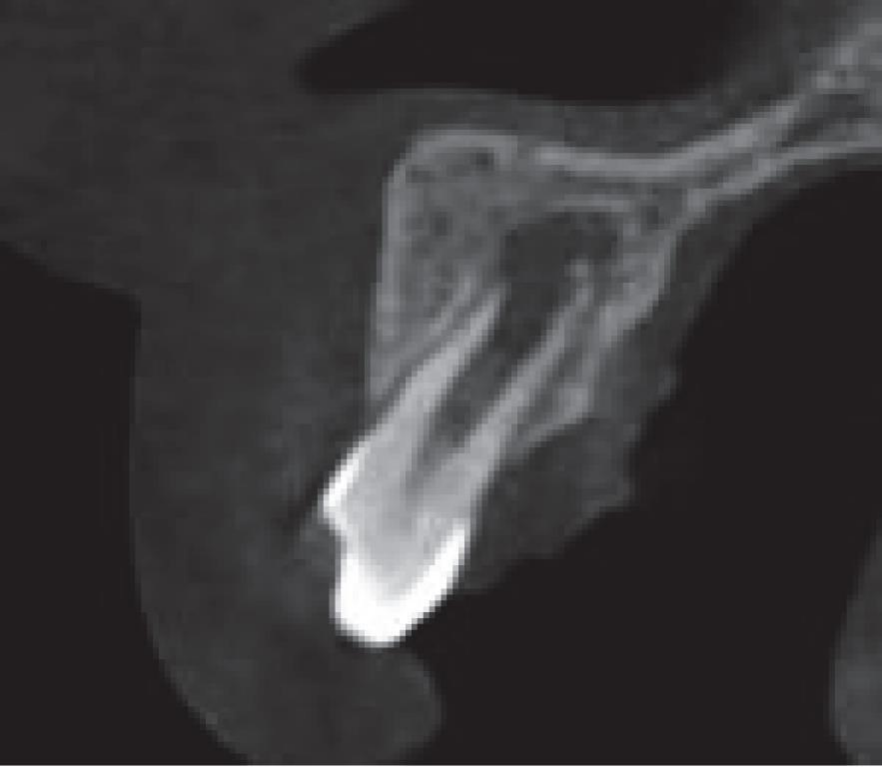
(C)

**Figure:**
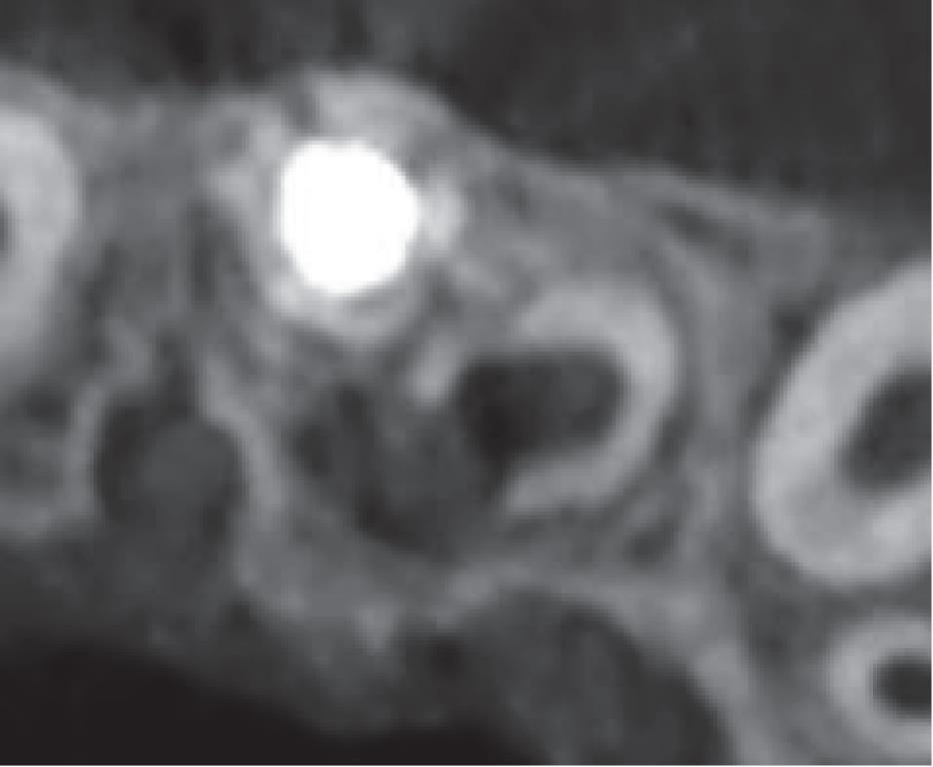
(D)

#### 2.1.1. Treatment of Tooth #10 With REP

Since the traditional pulp sensibility tests were inconclusive, a dentin stimulation test (without anesthesia) was performed. A positive response to the cavity test (scratching of the exposed dentin/cementum) confirmed pulp vitality. At the first appointment, treatment began without local anesthesia to confirm pulp necrosis. Following isolation and access cavity preparation, pus discharge was observed, confirming the diagnosis. The lateral incisor was treated with a REP in accordance with the guidelines of the American Association of Endodontists [25]. The working length was determined radiographically using a #20 H‐file (Figure 3A). Necrotic tissue remnants were carefully removed with a Hedström file, avoiding apical root canal wall contact. The canal was irrigated with 20 mL of 1.5% sodium hypochlorite. The root canal was dried with sterile paper points, and double antibiotic paste with ciprofloxacin and metronidazole (1:1) was used as an intracanal medicament. Finally, the access cavity was temporarily sealed.

Figure 3(A) Initial file periapical radiograph. (B) Postoperative periapical radiograph of tooth #10, restored with a 3‐mm composite resin restoration (immediate post‐treatment).

**Figure:**
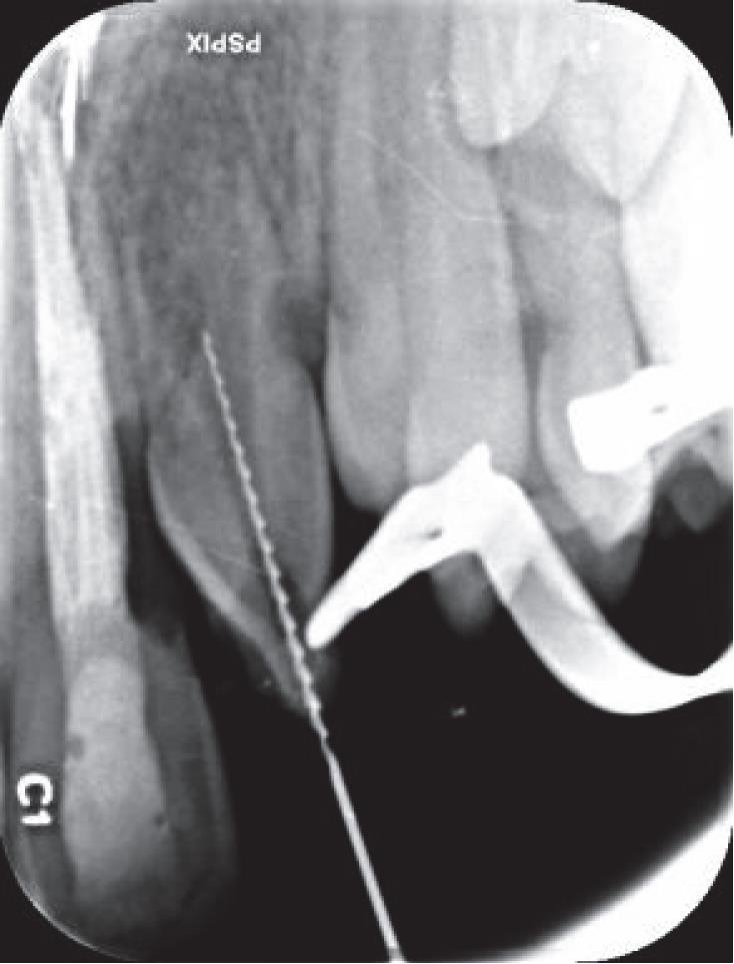
(A)

**Figure:**
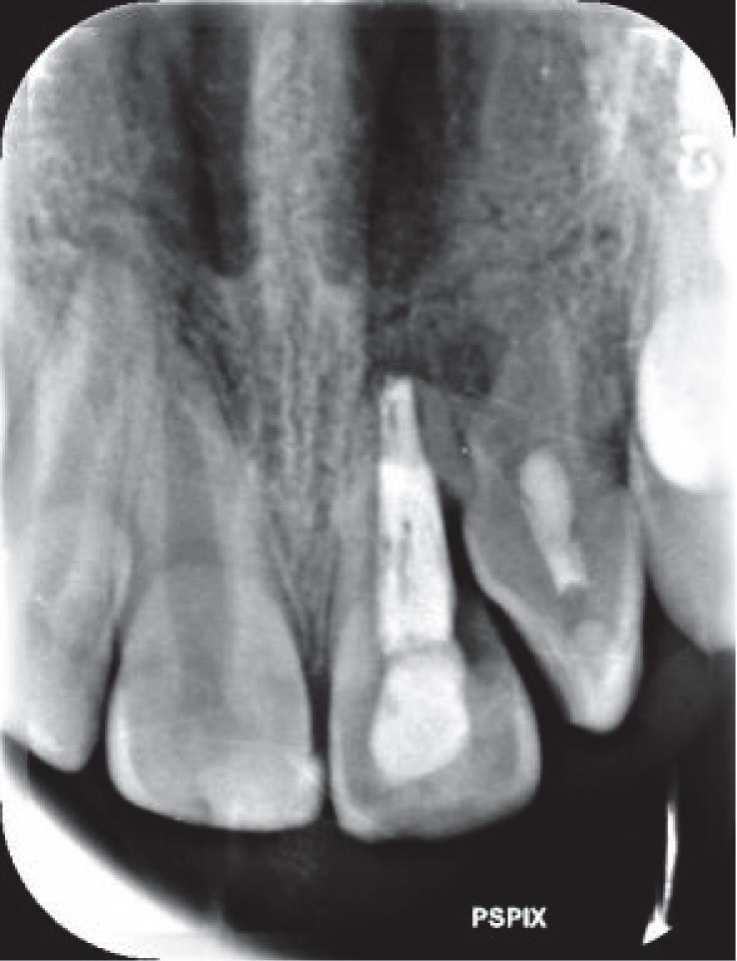
(B)

At the 3‐week recall, the patient remained asymptomatic. Platelet‐rich fibrin (PRF) was prepared from 10 mL of venous blood drawn from the right cubital fossa using a butterfly needle and centrifuged (1300 rpm, 8 min). Following anesthesia induction (with 3% mepivacaine) and rubber dam isolation, the temporary restoration was removed. The canal was irrigated with 20 mL of 17% EDTA, dried, and PRF was applied up to the cementoenamel junction using pluggers. A resorbable collagen plug (Lyostypt, Braun, Spain) was placed over the PRF and covered with NeoMTA2 (Avalon Biomed, USA). The coronal seal was completed with the application of glass ionomer cement (Fuji IX, GC Corp, Japan). After 7 days, the tooth was restored with a 3‐mm‐thick composite resin (Figure 3B). Follow‐ups were scheduled. The tooth remained asymptomatic after the completion of REP. Clinical and radiographic follow‐ups showed repair of the apical closure and absence of signs and symptoms of inflammation. Also, at the 15‐month follow‐up, increased coronal development was observed in tooth #10 compared to baseline, demonstrating a successful REP outcome (Figure 4B). In accordance with the guidelines of the American Association of Endodontists [25], a CBCT scan was obtained from tooth #10, which revealed significant apical closure compared to baseline, with an intact PDL (Figure 4C).

Figure 4Intraoral photographs. (A) Pretreatment clinical presentation. (B) Coronal eruption of tooth #10 at 15 months. (C) A 15‐month postoperative CBCT sagittal view of tooth #10 following REP demonstrating apical closure and dentinal wall thickening.

**Figure:**
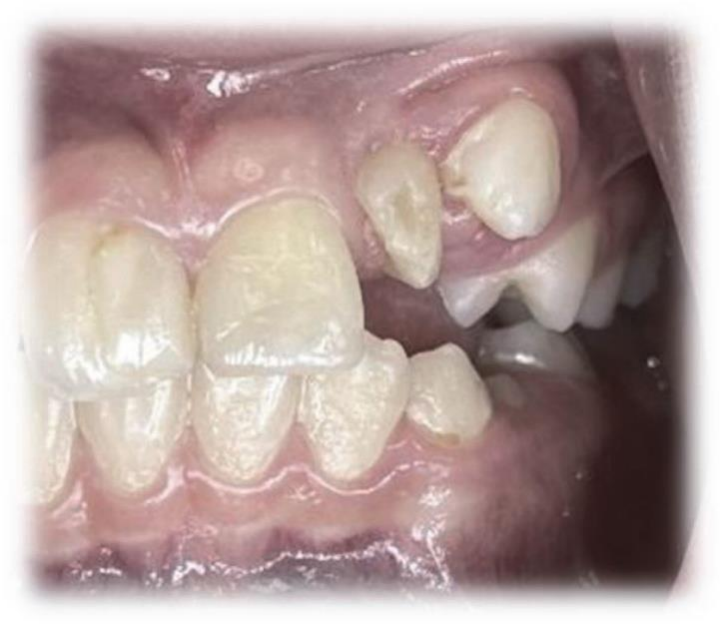
(A)

**Figure:**
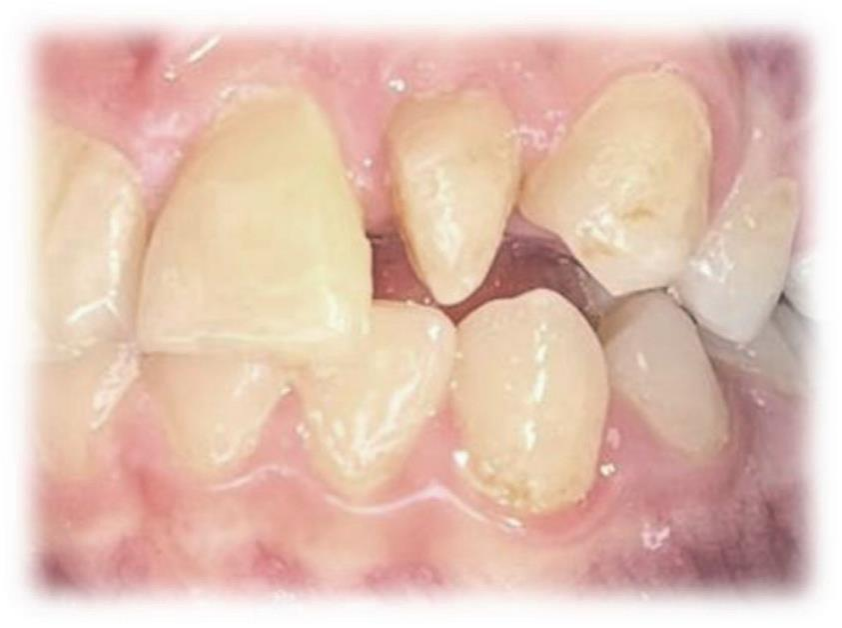
(B)

**Figure:**
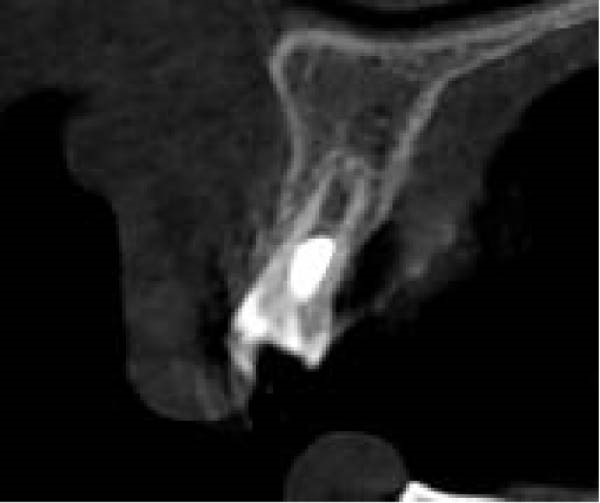
(C)

#### 2.1.2. Treatment of Tooth #9 With IR

**Figure 5:**
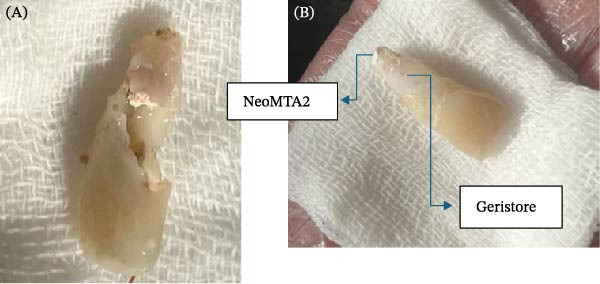
(A) Tooth #9 after granulation tissue removal. (B) Post‐reconstruction of the resorption defect with RMGI.

Local anesthesia was administered via an anterior superior alveolar nerve block using 4% articaine with 1:120,000 epinephrine (Darupakhsh, Iran). Supra‐alveolar fibers were circumferentially dissected, and the tooth was extracted atraumatically using conventional forceps (Dena Puya, Iran); elevators were avoided to preserve the PDL. The forceps were positioned firmly on the crown above the cementoenamel junction, and buccolingual luxation was slowly performed. Following extraction, the socket walls were carefully preserved to prevent PDL damage. The granulation tissue was removed with a small curette (Figure 5A).

The extracted tooth was held by the crown with a gauze dipped in saline. A 2‐mm apical root resection was performed, and a root‐end cavity was prepared using an ultrasonic tip. NeoMTA2 cement was placed as a retrograde sealing material, and excess material was removed with sterile gauze. The defect site was sealed with Geristore resin‐modified glass ionomer (RMGI) (DenMat, USA), and a RMGI restoration was applied and light‐cured using a blue light curing unit (PenCure 2000, Morita) (Figure 5B).

Prior to replantation, the apical socket was lightly curetted to remove the residual granulomatous tissue. The tooth was repositioned axially using digital pressure, and biting force aided in its final seating. Socket wall compression ensured optimal adaptation, after which the tooth exhibited primary stability but was splinted to the adjacent teeth using an archwire and composite resin (Figure 6). Occlusal adjustments were made to eliminate contacts. The extraoral working time for the IR procedure was 8 min.

Figure 6Tooth #9 was splinted to the adjacent teeth. (A) Intraoral view. (B) Radiographic view.

**Figure:**
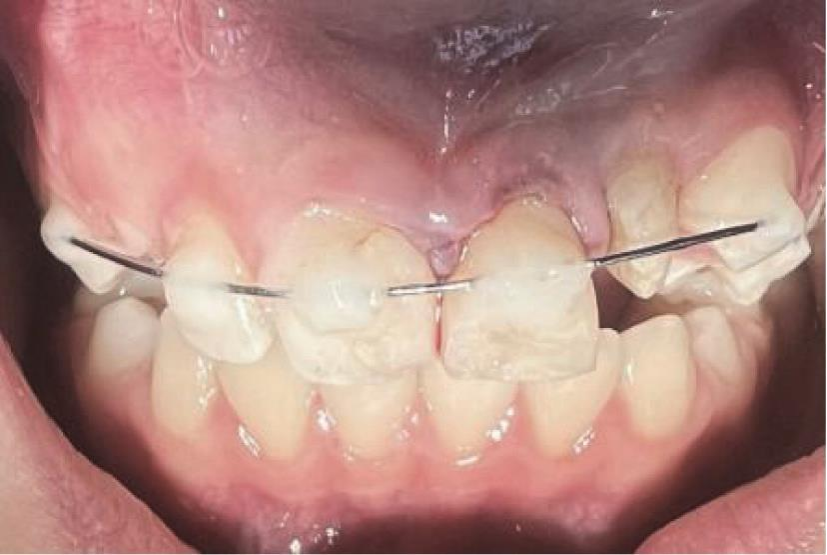
(A)

**Figure:**
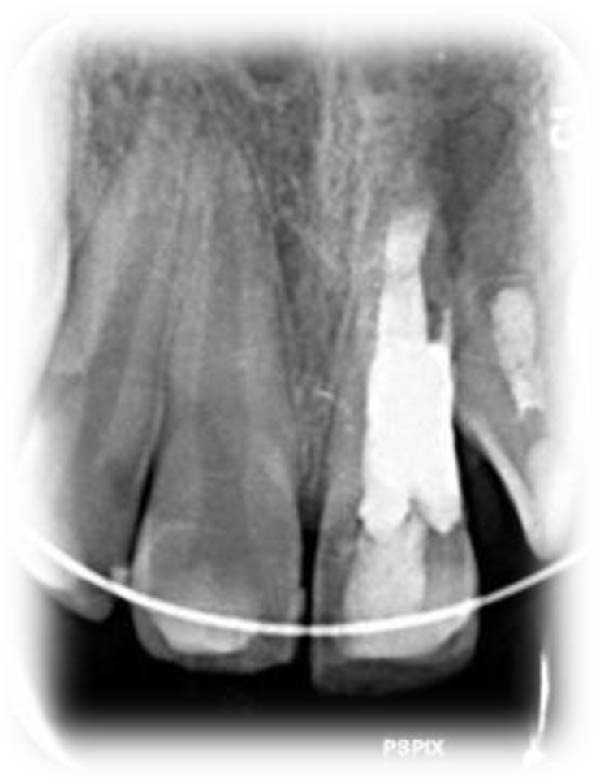
(B)

Postoperative instructions included 0.12% chlorhexidine gluconate rinse (Nikdarman, Iran) twice daily and a soft diet for 2 weeks. The semi‐rigid splint was removed after 4 weeks; at that point, the patient was asymptomatic, and tooth mobility was within the normal limits.

#### 2.1.3. Follow‐up

At the 3‐month follow‐up, both teeth were asymptomatic with normal clinical test responses. Radiographic examination revealed no progression of resorption in tooth #9, with evidence of periradicular healing (Figure 7C). Tooth #10 exhibited no change in root width or length.

Figure 7(A) Pretreatment, (B) Post‐IR, (C) 3‐month follow‐up, and (D) 15‐month follow‐up, demonstrating the initiation of root ankylosis on the mesial surface and coronal extrusion of the tooth #9.

**Figure:**
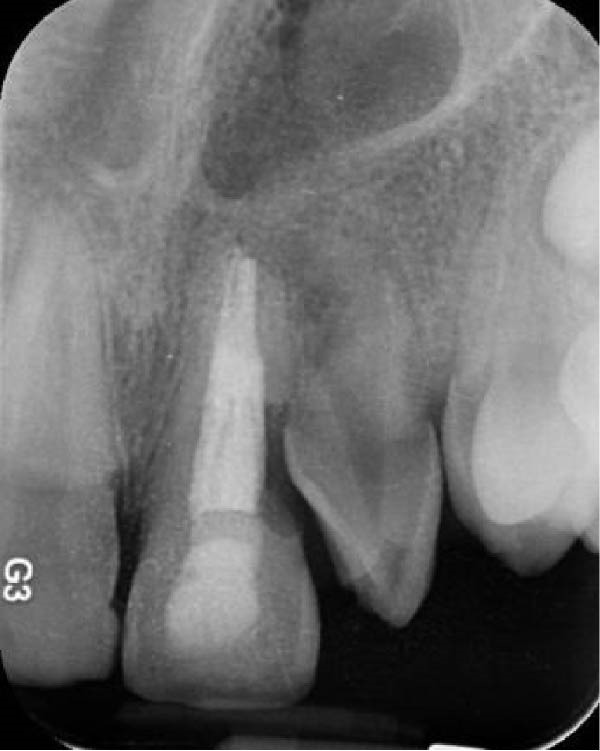
(A)

**Figure:**
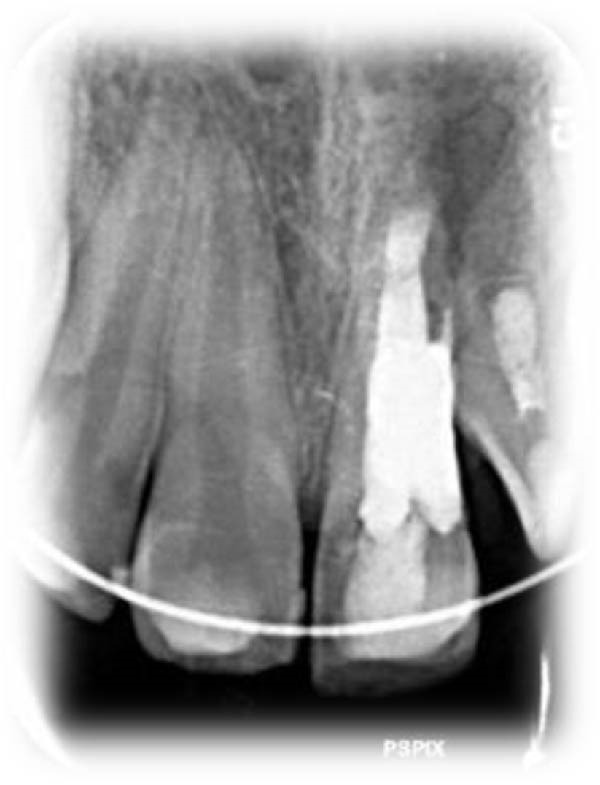
(B)

**Figure:**
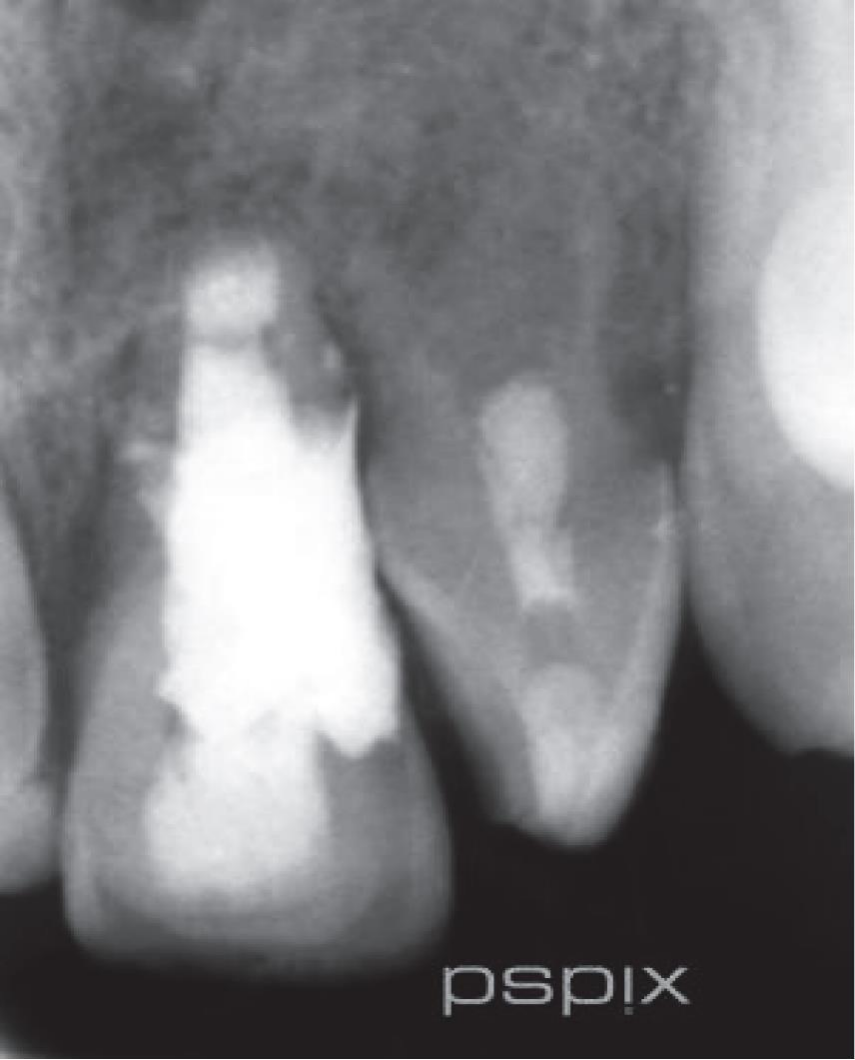
(C)

**Figure:**
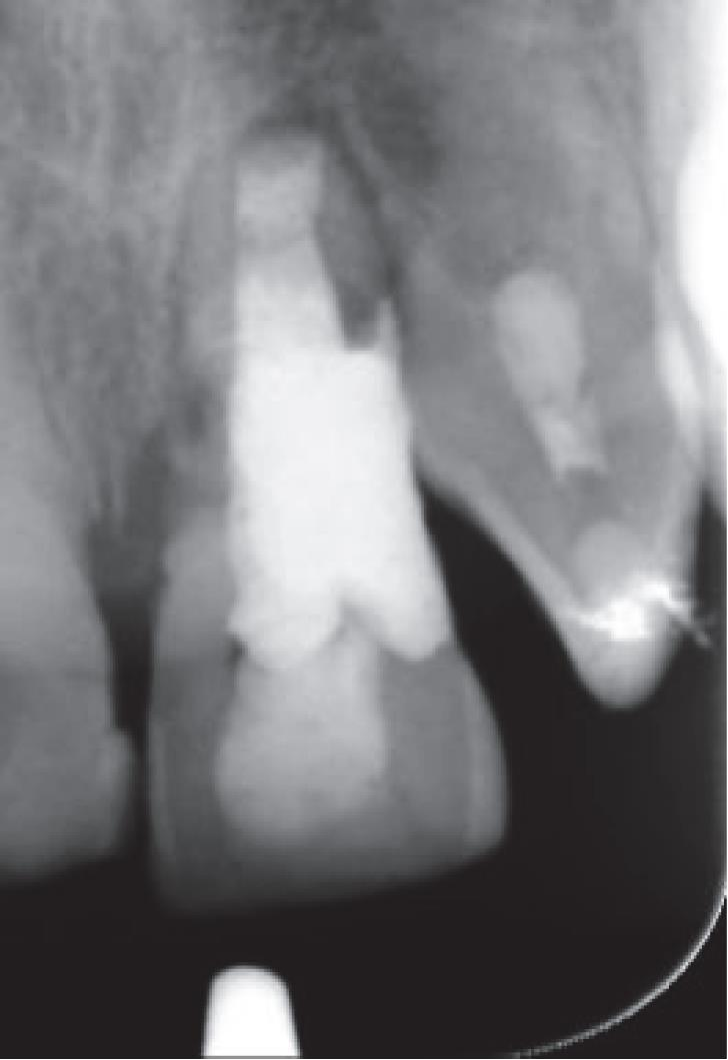
(D)

At the 15‐month follow‐up, both teeth remained asymptomatic. However, radiographs demonstrated continued resorption in tooth #9, including newly observed replacement resorption on the mesial surface (Figure 7D). This finding reflects compromised PDL healing and is consistent with the poor prognosis of IR in teeth with necrotic or severely damaged PDL, particularly following avulsion injuries. Although this outcome cannot be considered a biological success, the temporary retention of the tooth provided functional and esthetic benefits during a critical growth period. Accordingly, this outcome should be characterized as temporary functional preservation rather than definitive therapeutic success.

### 2.2. Case 2

A 30‐year‐old male patient presented with discomfort and pain in the right anterior maxilla. His dental history revealed trauma approximately 10 years earlier, multiple jaw surgeries, and root canal treatment of teeth involved in the fracture line. His medical history was noncontributory. Clinical examination revealed grade 1 mobility in teeth #7 and #8 without pathological mobility in the adjacent teeth. Palpation of the maxillary anterior region was normal, but mild sensitivity in percussion was noted in teeth #7 and #8. Pulp sensibility testing (cold test [Luber Cold Spray]) showed no response in teeth #6, #7, and #8. Radiographic evaluation (panoramic and periapical) identified ECR in tooth #8 and apical radiolucencies in teeth #7 and #8 (Figure 8A,B). The treatment plan consisted of nonsurgical retreatment of teeth #7 and #8, primary root canal therapy for tooth #6 (Figure 8C), and management of tooth #8’s cervical resorption via an internal approach using mineral trioxide aggregate (MTA) restoration (Figure 8E). At the follow‐up visits, the patient reported complete resolution of symptoms in the right anterior maxilla. All procedures were performed by an endodontist in private practice.

Figure 8(A) Panoramic radiograph. (B) Periapical radiograph demonstrating ECR of tooth #8. (C) Cone fit radiograph. (D) Immediate postoperative photograph. (E) Post‐treatment radiograph confirming MTA obturation of the ECR defect via internal access.

**Figure:**
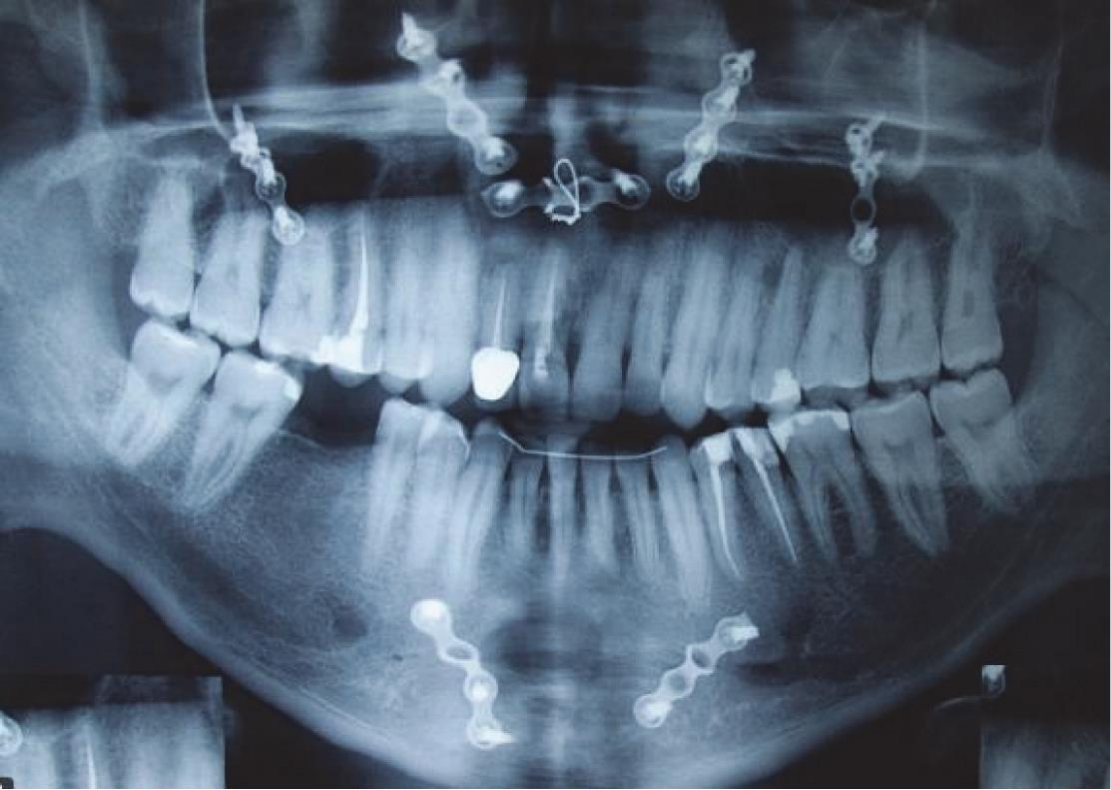
(A)

**Figure:**
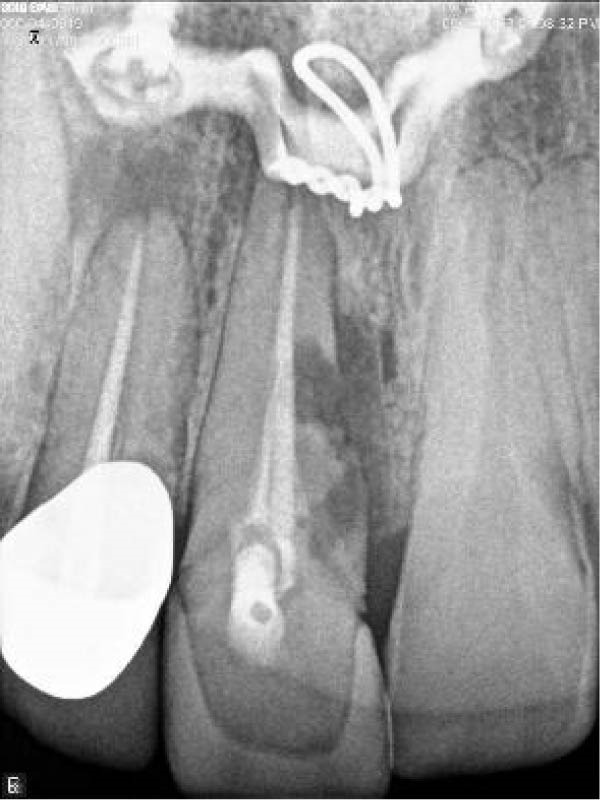
(B)

**Figure:**
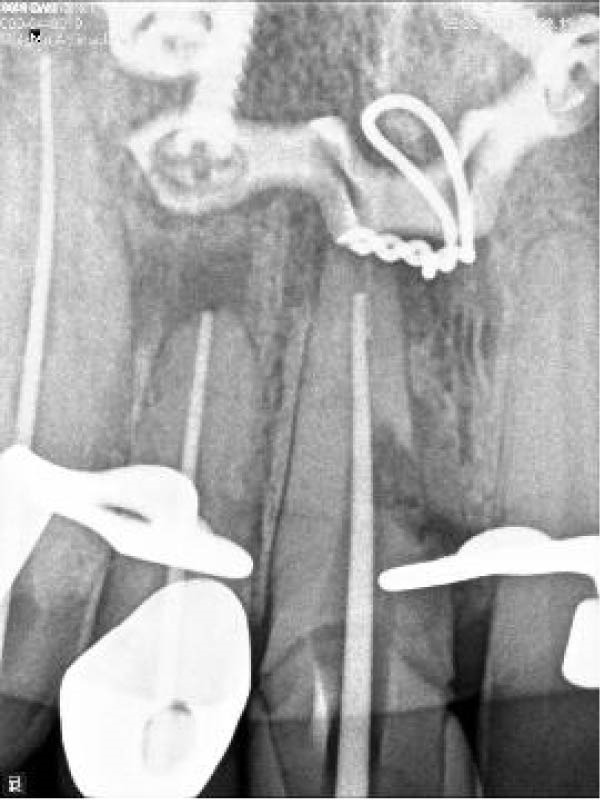
(C)

**Figure:**
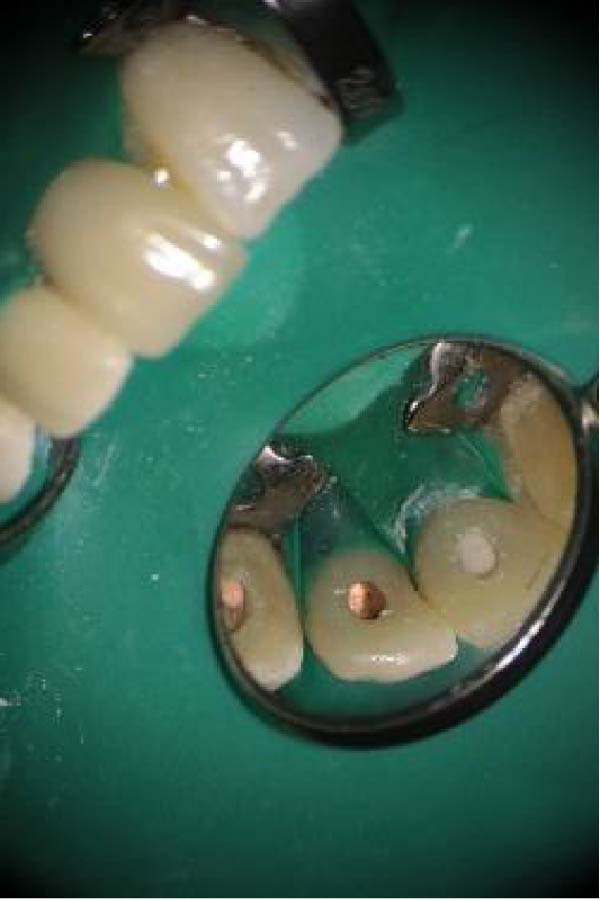
(D)

**Figure:**
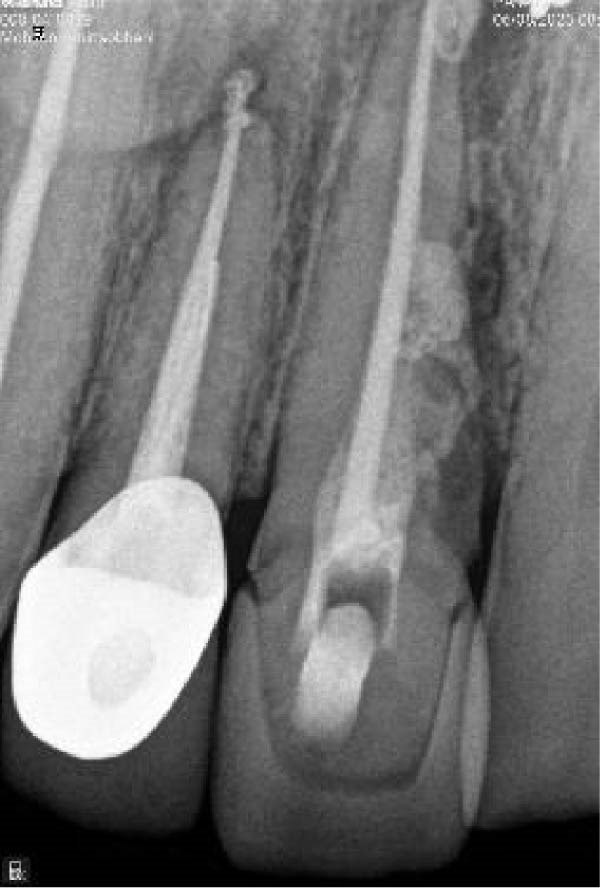
(E)

Three years later, he returned to the Endodontics Department of the Faculty of Dentistry for a routine examination, reporting altered sensation in tooth #8. During the initial visit, the right maxillary central incisor exhibited slight pain upon percussion and palpation, while the adjacent teeth were asymptomatic. Both the right central and lateral incisors demonstrated grade 2 mobility, with no pathological mobility in the adjacent teeth. Periapical radiographs revealed progressive ECR in the central incisor, extending mesially with associated radiolucency along the root surface (Figure 9A). A sinus tract was traced from the gingival sulcus of the central incisor, and a probing depth of 5 mm was recorded on the mesiofacial and mesiopalatal aspects. To evaluate the extent of the cervical resorptive lesion and facilitate precise classification and treatment planning, CBCT was performed (Figure 9B,C). Sagittal and axial CBCT images demonstrated extensive vertical (involving one‐third to one‐half of the root) and horizontal (~180°, predominantly palatal) expansion of the ECR lesion. Based on the Heithersay and Patel’s classifications, the lesion was diagnosed as Class IV and 3Bd, respectively [18, 19]. Following diagnosis and classification, the treatment options were discussed with the patient. Due to the extent of ECR, nonsurgical root canal retreatment was deemed unfeasible without concomitant periodontal surgery, including debridement and restoration of the affected hard tissue. After a thorough explanation, the patient expressed a strong preference for tooth retention, and informed consent was obtained for IR. Alternative options—including extraction followed by a conventional bridge, adhesive bridge, or dental implant (immediate or delayed)—were also discussed. The advantages and disadvantages of each approach were thoroughly reviewed before proceeding.

Figure 9Intraoral radiograph and CBCT. (A) Periapical radiograph revealing a detectable mesial root surface defect. (B) Sagittal CBCT demonstrating lesion extension beyond the cervical third of the root. (C) Axial view.

**Figure:**
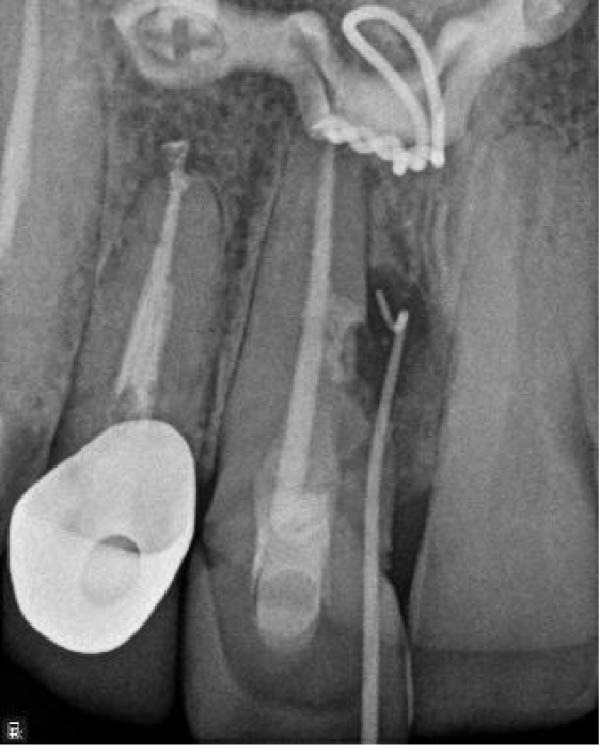
(A)

**Figure:**
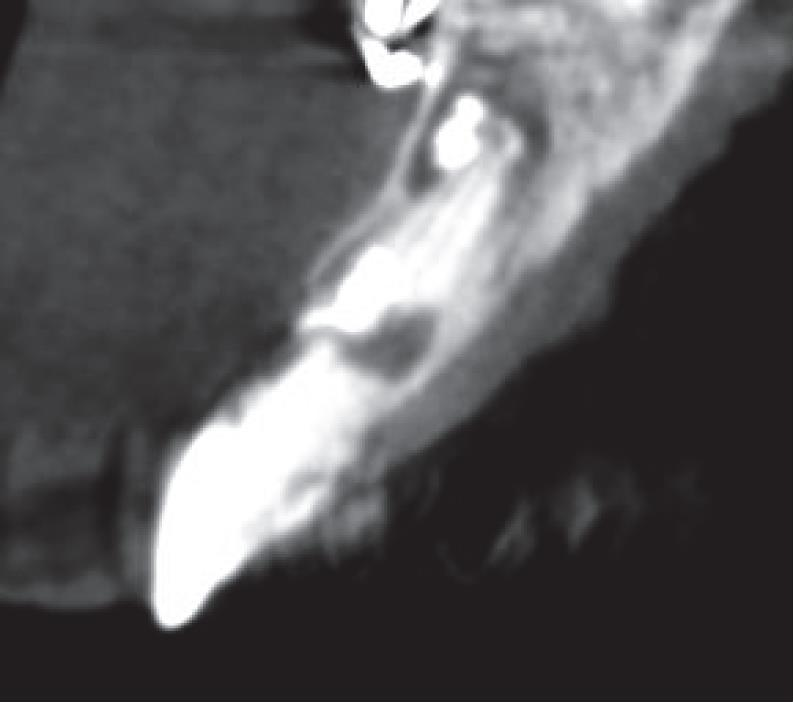
(B)

**Figure:**
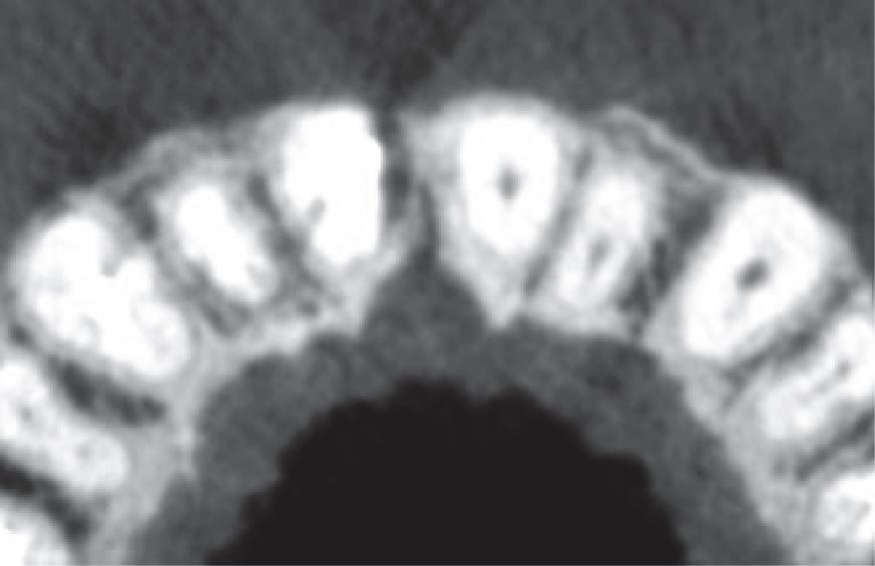
(C)

Based on the clinical findings, endodontic management of ECR was performed under magnification using a dental operating microscope (Labomed, USA). Preoperative disinfection of the oral cavity was achieved with 0.2% chlorhexidine (Nikdarman, Iran) rinse for 1 min, which was followed by induction of infiltration anesthesia using 2% lidocaine with 1:80,000 epinephrine (Darupakhsh, Iran).

Tooth #8 was atraumatically extracted using forceps (Dena Puya, Iran), allowing direct visualization and management of the ECR lesion under magnification. Following extraction, biopsies were obtained from the cervical and apical regions of the tooth‐associated soft tissues and processed for histopathological examination (Figure 10). The resorptive defect was meticulously curetted to ensure complete removal of the granulation tissue. A 3‐mm apicoectomy was performed perpendicular to the longitudinal axis of the tooth using a high‐speed truncated conical diamond bur (FG ML, Diatech, Coltène/Whaledent, Switzerland) under continuous water cooling.

Figure 10(A) Arrow points to a narrow strip of nonkeratinized junctional epithelium. (B) Arrow shows the erosive dentinal surface, indicating active resorption.

**Figure:**
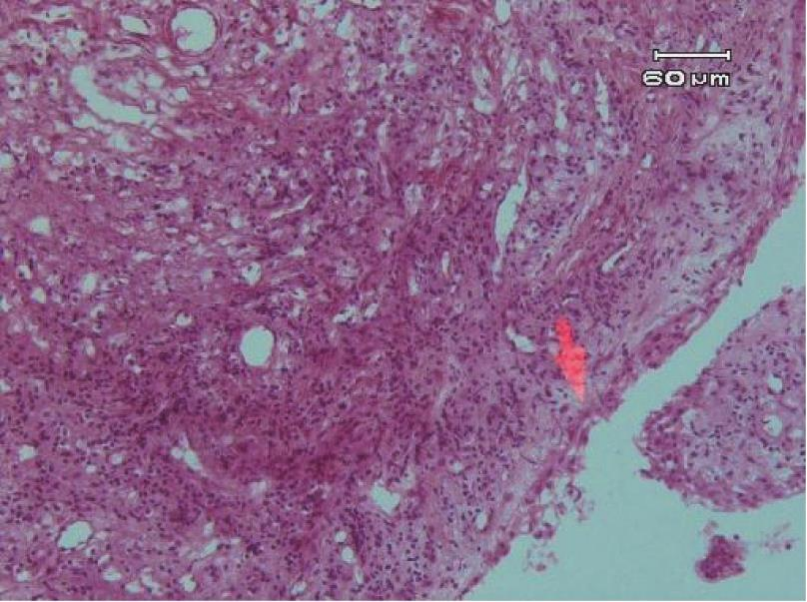
(A)

**Figure:**
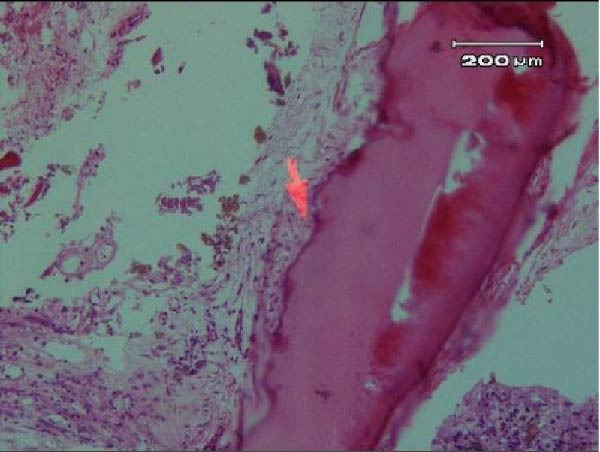
(B)

Apical preparation (3 mm depth) was performed using ultrasonic tips (E32D, NSK, Japan), followed by cavity drying and obturation with Edge MTA (Edge Seal; Hamerz, Iran). The gingival and coronal margins of the defect were restored with RMGI cement (Willmann & Pein GmbH, Germany), while the mid‐root and apical regions were restored with MTA (Edge Seal; Hamerz, Iran) (Figure 11).

Figure 11(A) RMGI placement. (B) MTA placement in the mid‐root region.

**Figure:**
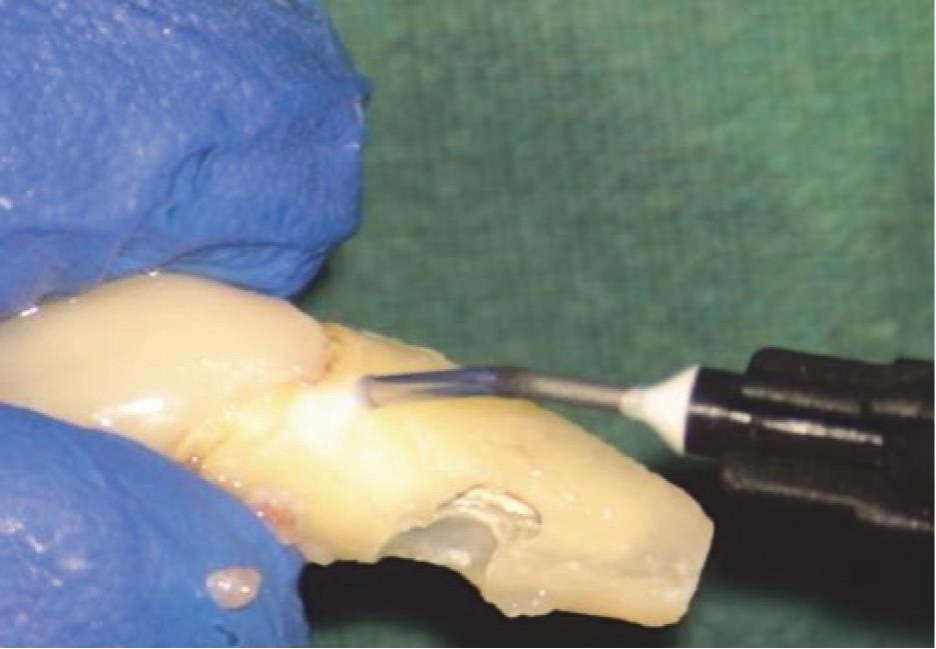
(A)

**Figure:**
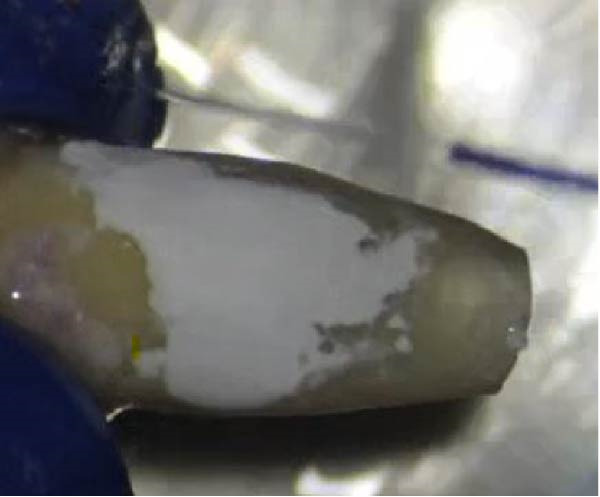
(B)

The tooth was then replanted into the socket. A sterile saline solution was used to prevent desiccation and contamination throughout the procedure, except during ECR removal and RMGI/MTA restoration modifications (performed with a high‐speed handpiece). To enhance initial stability and wound healing, the crown was sutured to the adjacent teeth (Figure 12A,B). The total extraoral time was limited to 10 min. Follow‐up evaluations were conducted at the scheduled intervals. At the 2‐week recall, the sutures were removed and the tooth remained asymptomatic with no clinical abnormalities (Figure 12D). The postoperative period was uneventful. At the 6‐month follow‐up, radiographic and clinical examinations revealed that tooth #8 was in normal occlusion, exhibited physiological mobility, and had effective masticatory function. Periodontal probing detected no pathological pockets, and the patient remained asymptomatic and satisfied with the outcome. Radiographic evaluation 6 months and 18 months postoperatively demonstrated bone formation, with no evidence of root resorption or periapical pathosis (Figure 13C,D).

Figure 12(A, B) Clinical photograph of the tooth splinted to adjacent teeth. (C) Immediate postoperative radiograph following IR. (D) Postoperative view at 2 weeks after removal of the splint suture, revealing stable tissue contours and successful initial healing phase.

**Figure:**
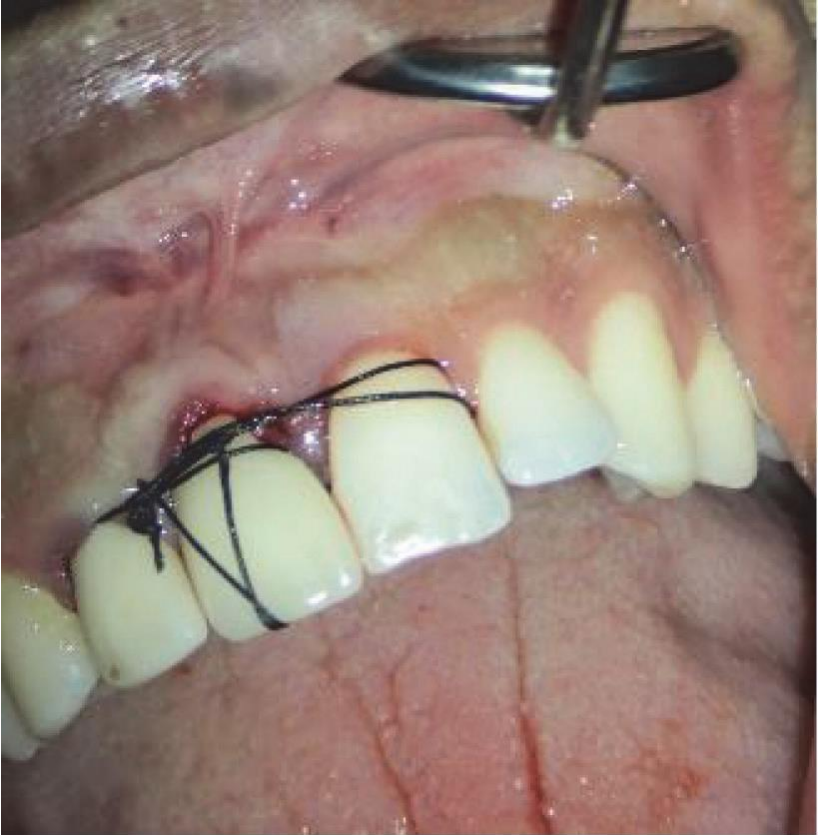
(A)

**Figure:**
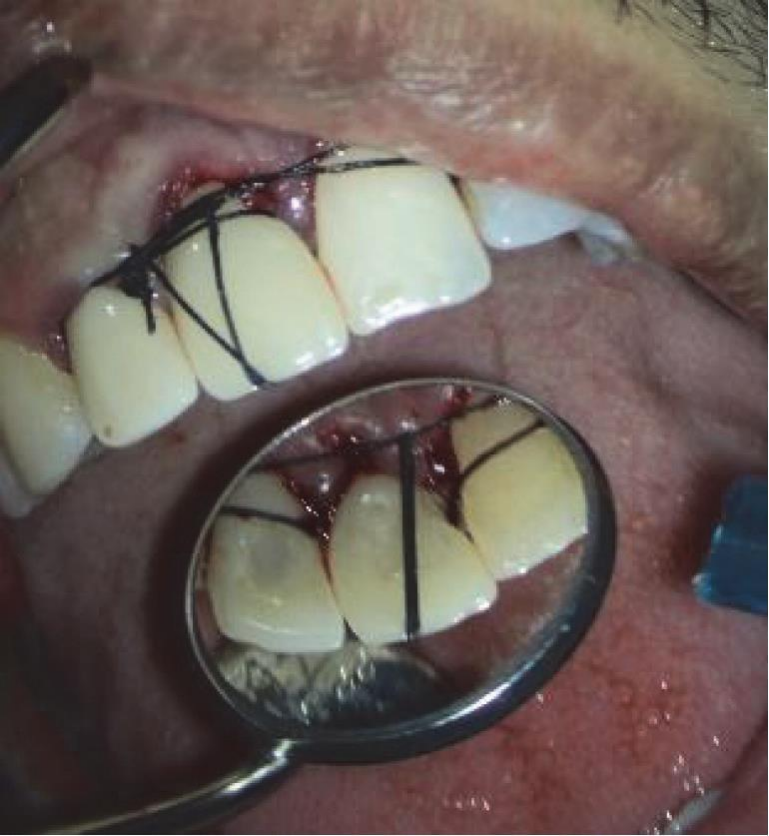
(B)

**Figure:**
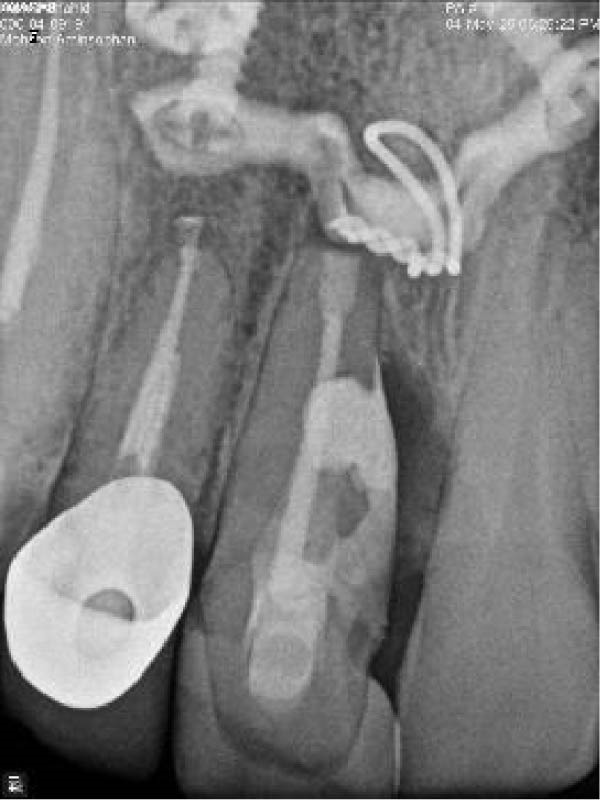
(C)

**Figure:**
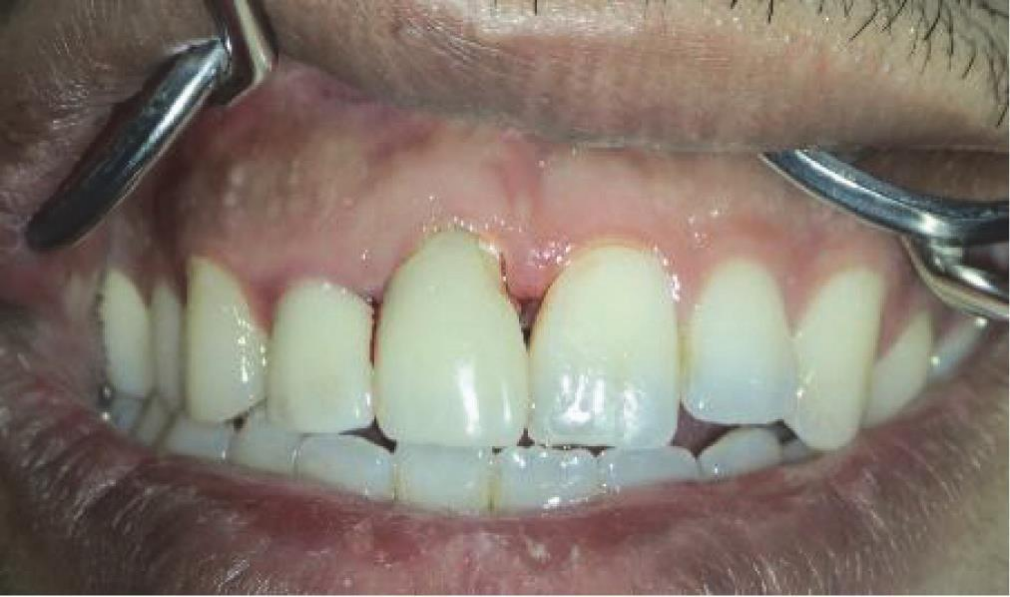
(D)

Figure 13Radiographic sequence of the management of ECR. (A) Pre‐operative presentation of the resorptive lesion. (B) Immediate postoperative radiograph following IR. (C) At the 6‐month review, radiograph confirmed successful healing, characterized by bone formation and regeneration of the alveolar bone. (D) At the 18‐month follow‐up, the maxillary right central incisor demonstrated radiographic evidence of new bone formation in the mesial middle third, and the patient remained clinically asymptomatic.

**Figure:**
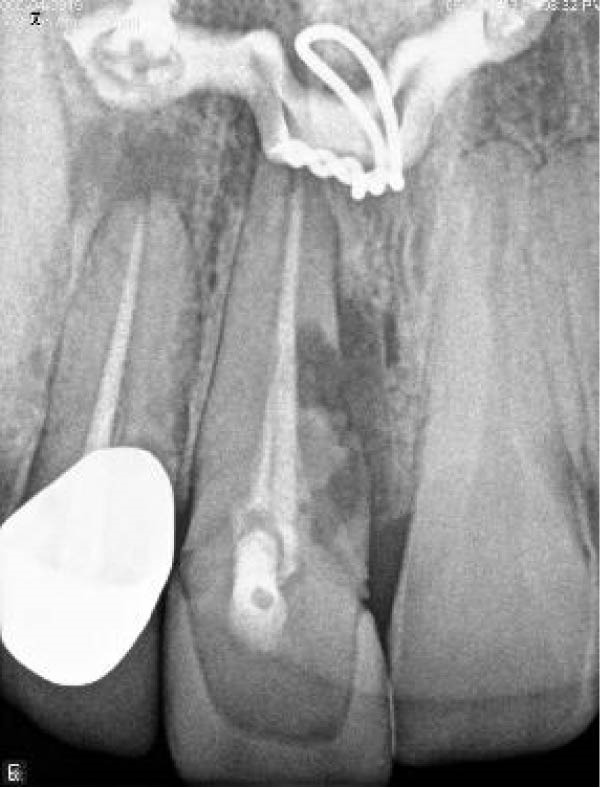
(A)

**Figure:**
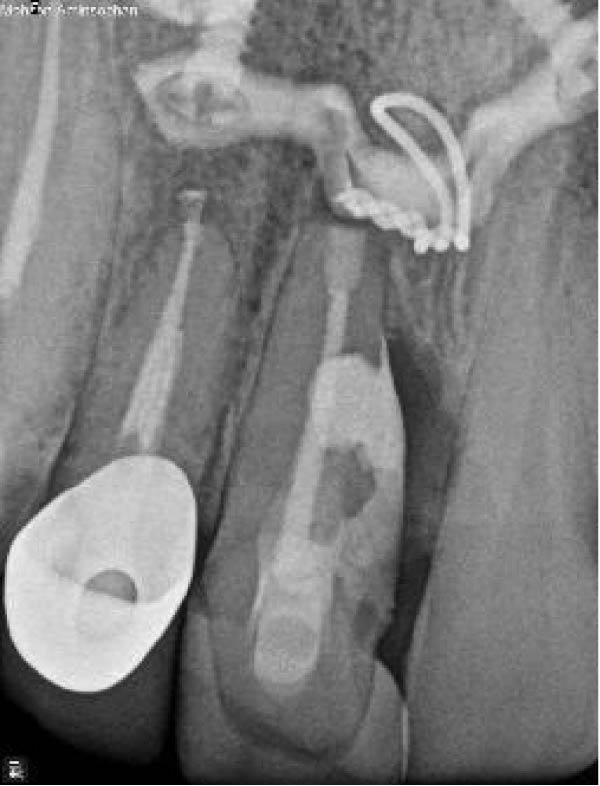
(B)

**Figure:**
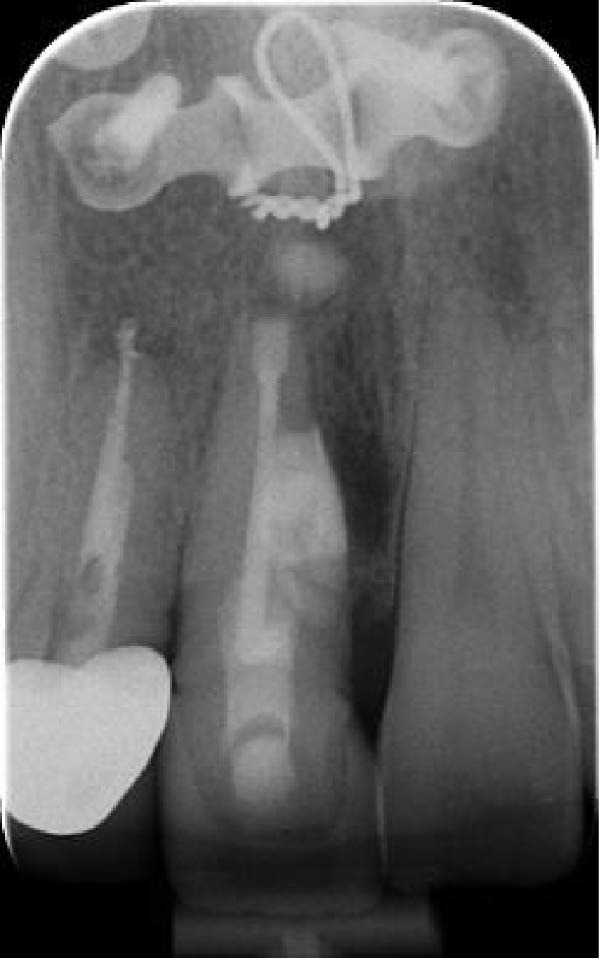
(C)

**Figure:**
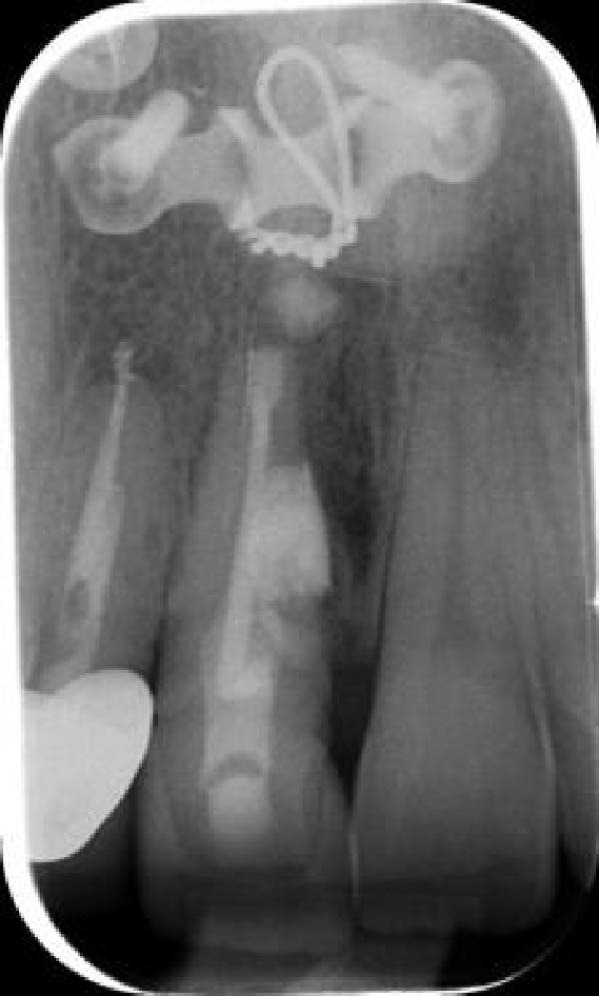
(D)

## 3. Discussion

Dental traumatic injuries are prevalent in children and young adults, constituting 5% of all body injuries [26]. Permanent tooth avulsion is among the most severe dental injuries, although not the most common. Prompt, appropriate emergency management is crucial, as prognosis heavily depends on adherence to avulsion guidelines. Under ideal conditions, replantation can preserve a functional tooth for decades. The tooth may heal completely, although pulp canal obliteration often occurs. Complications, including pulpal necrosis, ankylosis, root resorption (particularly ECR), excessive mobility, and arrested root formation in immature teeth with open apices [27].

Tooth loss at a young age is a distressing experience for both the patient and parents, with psychological impacts often surpassing the immediate physical effects [28]. Premature loss of permanent teeth can result in alveolar bone resorption and growth arrest, complicating future esthetic and functional rehabilitation [29]. Consequently, the International Association of Dental Traumatology guidelines stress the importance of preserving permanent teeth whenever possible [30].

ECR is related to traumatic injuries, luxation, and avulsion in particular, which locally damage the PDL [27]. The occurrence of ECR in such cases may be attributed to residual inflamed PDL cells or pulp tissue. Meticulous root surface and canal debridement may potentially prevent ECR.

Various treatment options have been suggested for managing teeth with ECR. The European Society of Endodontology Position Statement outlines potential treatments, including external repair, internal repair, IR, periodic monitoring, and extraction [31]. Similarly, a recent review discussed treatment modalities, such as external repair, internal repair, palliative care, and extraction [32]. However, no standardized protocol exists for ECR management, nor have globally accepted guidelines been established to correlate specific treatment approaches with clinical scenarios [1]. In Case 1, implant‐supported restorations were contraindicated due to the patient’s age, and fixed partial dentures could present adaptation challenges. In both cases, partial dentures were not a suitable treatment option due to the risk of aspiration, making the situation challenging, and they were not recommended. Alternatively, tooth extraction with orthodontic space closure could be considered; however, this approach may lead to asymmetry and may require modification of the adjacent sound teeth [33]. According to the European Society of Endodontology Position Statement, IR was selected as the treatment of choice for both cases to allow restoration and/or recontouring of an otherwise inaccessible resorption defect [31].

Several pre and perioperative prognostic factors influence the success of IR. The key preoperative risk factors include the presence of periodontal pockets ≥ 6 mm and patient’s age > 40 years. The integrity and vitality of the PDL are widely recognized as the most critical determinants of post‐replantation healing. However, despite its prognostic importance, no clinically reliable method currently exists to objectively assess PDL vitality prior to tooth extraction. Clinical parameters, such as tooth mobility, probing depth, percussion sensitivity, and radiographic appearance of the periodontal space, provide only indirect information and cannot determine cellular viability. Consequently, PDL status is often inferred retrospectively based on healing patterns observed during follow‐up. In Case 1, although preoperative clinical findings did not indicate periodontal breakdown, the history of avulsion with prolonged extraoral dry time strongly suggested severe PDL compromise. Conversely, perioperative factors that improve the outcome include atraumatic extraction, piezoelectric root resection, preservation of cellular and acellular cementum, extraoral handling time ≤ 15 min, application of bioactive root‐end filling materials, and magnification use. These findings underscore the importance of adhering to modern microsurgical endodontic principles to optimize IR prognosis [34]. Furthermore, significant variability has been observed in root resection techniques, obturation materials, and socket management, while resection length has been infrequently reported, with most studies reporting a mean of 1–3 mm [35]. The commonly used restorative materials for this purpose include dental amalgam, contemporary bioceramics (e.g., MTA, SuperEBA, and Endocem), and zinc oxide‐eugenol or glass ionomer cements. Alveolar manipulation techniques vary, ranging from simple clot aspiration and saline irrigation to mechanical curettage—the latter posing risks of PDL damage, potentially compromising the IR outcomes. These findings underscore the critical influence of socket handling on IR success, which remains a contentious topic in endodontics [23].

This study selected IR as the treatment of choice due to several factors: preserving esthetics and inducing bone formation, operator confidence, favorable intraoral access, coronal tooth structure preservation, and adequate orthograde root canal treatment. Additionally, periapical surgery is often impractical. Patient‐related factors, such as financial constraints and a preference for tooth retention over extraction, also influenced this decision. Importantly, in the context of advanced ECR, IR should be regarded primarily as a palliative or temporizing strategy rather than a definitive biologically curative treatment, with the primary objective of delaying extraction while maintaining function, esthetics, and alveolar bone. The ability of this technique to allow complete extraoral visualization and management of the resorptive defect without damaging adjacent tissues supports its use in carefully selected cases for short‐term functional preservation [36].

Mavridou et al. [37] discussed how the treatment mechanism in endodontically‐treated teeth was similar to that in vital teeth, with a key difference observed at the entry site. Specifically, endodontically‐treated teeth exhibited no invasion into the adjacent bone tissue or fusion with tooth structure, likely due to the absence of vital pulp tissue. Vital pulp regulates oxygen tension and mitigates local hypoxia within the resorption cavity. Hypoxia is a critical factor in ECR progression in both vital and endodontically treated teeth, promoting angiogenesis and sustaining the highly vascular granulation tissue, characteristic of ECR [38]. Previous studies have demonstrated similarities between endodontically‐treated teeth and vital teeth. However, a key difference is the significantly greater resorption intensity observed in endodontically‐treated teeth, characterized by numerous multinucleated cells, active tooth structure resorption, and extensive resorption extending to the root canal filling material [37]. This more aggressive form of invasive root resorption may occur due to the following factors:

1. Absence of a vital pulp and a pericanalar resorption‐resistant sheet, which is a mineralized layer surrounding the vital pulp that protects against clastic cell invasion, with its resistance attributed to pulp vitality or its inherent mineralization gradient [10, 37].
2. Root canal irrigants like NaOCl, EDTA, citric acid, and H_2_O_2_ that can alter the root dentin’s chemical composition, particularly affecting its matrix and protein content, which may disrupt bone cell activity [39–41].

In the current cases, elevators were not used to avoid tooth fracture, and the extraoral time was limited to 10 min. Resorptive tissues were removed under magnification with copious irrigation. Also the selection of 10% citric acid for root conditioning in our IR cases is supported by its ability to demineralize dentin and expose collagen, which may facilitate PDL fibroblast attachment [42]. However, this approach remains controversial, as EDTA—with its neutral pH and chelating properties—is considered a less aggressive alternative, while saline maintains hydration without active conditioning. No consensus exists regarding the optimal agent, as comparative clinical studies in IR are lacking. Our use of citric acid reflects reported protocols rather than definitive evidence. Previous studies indicate that inadequate root‐end filling material and insufficient depth can contribute to IR failure. The success of IR also depends on an effective coronal and apical seal, which prevents reinfection, along with the biomaterial’s ability to promote healing [43]. The reason behind using RMGI in both cases was because of the fact that RMGI combines the benefits of traditional glass ionomer cement with light‐cure resin, offering improved properties such as enhanced adhesion, fluoride release, and optimal esthetics. RMGI also allows for rapid light‐activated hardening, making it a practical choice for clinical applications where strength and durability are required [2]. In Case 2, MTA was used to repair a mid‐root resorption defect due to its well‐documented bioactive properties, including its ability to promote dentinal bridge formation. It induces reparative dentinogenesis more effectively than calcium hydroxide, with superior structural integrity and less pulp necrosis. These advantages made MTA the preferred material to ensure optimal healing in this case [2].

Although literature reports high survival rates, unsuccessful IR does not compromise—and may even enhance—hard and soft tissue conditions for future extraction and prosthetic rehabilitation and with respect to PDL vitality, a success rate over 88% has been reported [27]. Thus, IR remains a viable treatment option that should be considered in patient discussions [44]. The suboptimal long‐term outcomes in this study may be attributed to extensive PDL damage during treatment, consistent with previous findings. Studies report a higher incidence of inflammatory resorption when over 25% of the PDL is compromised and when a narrow alveolus leads to direct tooth‐bone contact [33, 45]. In Case 1, due to the prolonged extraoral duration of the avulsed maxillary left central incisor and the extensive degenerated granulomatous tissue observed during treatment, the PDL cells were nonviable, and the PDL tissue was entirely necrotic.

Also, Case 1 involved a hard tissue defect extending from one‐third to half of the root length on the palatal side. Clinical and radiographic findings suggested that managing this advanced ECR defect would be challenging. Although IR is a reliable treatment for ECR, complications such as crown/root fracture during extraction, and PDL damage during ECR‐affected dentin removal, granulation tissue debridement, or adhesive restorative procedures may lead to external resorption or ankylosis. At the 3‐month recall, the patient was asymptomatic with no probing depth > 2 mm. By the 15‐month recall, although resorption was evident on the mesial surface of tooth #9, the primary goal of arresting further resorption and maintaining the tooth’s function and esthetics was achieved. As this study showed, while Case 2 demonstrated short‐term periodontal stability, Case 1 represents a clear example of functional survival without biological success, underscoring the importance of interpreting IR outcomes in advanced ECR as interim and case‐dependent rather than predictable long‐term biological solutions. Also in the context of IR, it is important to distinguish biological success, defined by stable periodontal healing without ankylosis or resorption, from survival or temporization, which describe continued short‐term tooth function despite ongoing pathological processes. In the present report, Case 1 reflects survival and temporization rather than biological success.

Research on the re‐eruption of young permanent teeth explores hydrostatic pressure and PDL traction, yet the optimal approach—whether spontaneous re‐eruption or active repositioning—remains [46, 47]. Spontaneous re‐eruption is more likely in children under 9 years, while orthodontic or surgical intervention depends on age, root development, and intrusion severity [26, 48]. In Case 1, the dentist initially monitored the tooth for spontaneous eruption for 4 months. When this approach failed, forceps repositioning was attempted, which may have contributed to necrosis in the immature tooth. Andreasen et al. [49] found that active repositioning in teeth with incomplete root formation increases the risks of pulp necrosis, resorption, and impaired bone healing. The International Association of Dental Traumatology recommends monitoring for revascularization before considering any intervention [50]. After repositioning, the tooth showed no signs of eruption or maturation, and necrosis was later confirmed. REP was performed, resulting in apex narrowing and root thickening at the 15‐month follow‐up, meeting the American Association of Endodontists’ success criteria [25].

A major limitation of the present report is the relatively short follow‐up period, which precludes conclusions regarding long‐term biological stability, ankylosis, or replacement resorption. In the management of ECR with IR, follow‐up of at least 3–5 years is generally recommended to reliably evaluate long‐term outcomes such as ankylosis, replacement resorption, and periodontal stability. Therefore, the outcomes presented here should be interpreted as intermediate, short‐term observations rather than definitive indicators of long‐term success. Future monitoring of these cases, along with studies incorporating longer observation periods, is necessary to draw conclusive evidence regarding the stability and prognosis of IR in advanced ECR.

## 4. Conclusion

The reported cases demonstrated that IR may serve as a short‐term, palliative option for selected cases of advanced ECR when conventional treatment or extraction is contraindicated. Given the limited follow‐up and the inherent biological risks, IR should not be interpreted as a predictable long‐term solution, but rather as a temporizing strategy aimed at delaying tooth loss while preserving function and esthetics.

## Funding

No funding was received for this manuscript.

## Ethics Statement

For clinical cases, the local ethics committee considers that the patient’s consent is sufficient.

## Consent

Written informed consent was obtained from the patient to publish this report in accordance with the journal’s patient consent policy.

## Conflicts of Interest

The authors declare no conflicts of interest.

## Data Availability

The data supporting the findings of the present study are available from the corresponding author upon request.
